# Medical and Philosophical Intelligence

**Published:** 1812-11

**Authors:** 


### MEDICAL AND PHILOSOPHICAL INTELLIGENCE.

PHILOSOPHICAL SOCIETY OF LONDON.—On Monday, October the 5th, being the Anniversary of the Philosophical Society of London, the members proceeded to ballot for the officers and council for the year ensuing, when the following gentlemen were appointed:

*President.*—J. C. Lettsom, M.D. LL.D. F.R.S.

*Vice-Presidents.*—Admiral Savage, J. Taunton, F.A.S., J. T. B. Beaumont, F.A.S., George Rees, M.D.

*Treasurer and Secretary.*—T. J. Pettigrew.

*Curators.*—Rev. Joseph Nightingale, Rev. James Andrewes.

*Registrar.*—J. Miers.

*Council.*—J. Adams, M.D. F.L.S., J. Sowerby, F.L.S., W. Bullock, F.L.S., J. Parkinson, F.L.S., E. Clarkson, T. Bedder, R. Thompson, J. Hare, W. Henley, B. Clarkson, E. Scargill, T. K. Cromwell, J. Hare, jun., J. Smither, H. Harper, J. B. Brown, W. Reid, R. Hone.

At half past three an annual Oration was delivered by Mr. Pettigrew, on the necessity of cultivating the mental powers, and the import of literary institutions, including an account of the origin and progress progress of the Society. We abstain from submitting an abstract of the Oration, as we understand it is the intention of the Institution to print it.

---

On Wednesday, October 7th, 1812, the Society for the Relief of Widows and Orphans of Medical Men in London and its Vicinity, held their Half-yearly General Court, at the usual place of meeting, the Gray's-Inn Coffee-house, Holborn, when the annual election of officers and directors took place, and the following were the arrangements made for the ensuing year.

*Patron.*—His Royal Highness the Duke of Kent.

*President.*—James Ware, Esq. F.R.S. &c.

*Vice-Presidents.*—Sir F. Milman, Dr. Lettsom, Dr. Blane, Dr. Squire, Dr. Dennison, Sir Wm. Blizard, Mr. Heaviside, Mr. Nevinson, Mr. Moore, Mr Rendall, Dr. Baillie, *vice* Dr. Garthshore, dec. Dr. Haworth, *vice* Mr. Howard, dec.

*Treasurers.*—Dr. Denman, Dr. John Sims, Mr. Upton, *vice* Dr. Dennison.

*Directors.*—Physicians, Dr. S. H. Jackson, Dr. R. Pearson, Dr. Marcet, Dr. Croft, Dr. Laird, Dr. Adams, Dr. Stone, Dr. Yelloly. Surgeons, Mr. H. L. Thomas, Mr. Ch. M. Clark, Mr. Ramsden, Mr. Lewis, Mr. Thomas Blizard, Mr. Chevalier, Mr. Ring, Mr. John Wilson. Apothecaries, Mr. Starr, Mr. Malim, Mr. Moore, jun. Mr. Pilliner, Mr. Wheeler, Mr. Hunter, Mr. Beaumont, *vice* Coates, Mr. Field, *vice* Seaton.

*Secretary.*—Mr. Wm. Chamberlaine.

*Collector.*—Mr. George Hunt.

This Society was instituted in the year 1788. Its capital is now Nineteen Thousand Two Hundred Pounds Three per Cent. Consolidated Bank Annuities, and Two Hundred Pounds Navy Five per Cents, out of the interest of which, down to the 16th of September in the present year, 1812, the sum of Three Thousand Five Hundred and Thirty-seven Pounds Thirteen Shillings has been distributed among the widows and orphans of deceased medical men, members of this Society, many of whose families have been left without any provision whatever.

The Anniversary Festival of this Society was celebrated on Thursday, October 29th, at the Freemasons' Tavern, his Royal Highness the Duke of Kent, in the Chair.

---

The Harveian Oration at the Royal College of Physicians, was delivered, on the 19th of October, by *Dr. Ash.* Dedicated, as this oration chiefly is, to the commemoration of the founders and benefactors of the College, and to the great luminaries of the medical art, much of novelty cannot annually be expected, unless every year presented some great discovery, or produced some extraordinary genius. On so hackneyed a subject, little more to interest the hearer can be now expected, than felicity of language and the grace of elocution. In these, Dr. Ash has not yielded the palm to his predecessors. It may require, however, to be particularly noticed, that this oration contained a warm panegyric on the practice tice of vaccination, and the praise of Dr. Jenner for his services to the public, which the author thought he so amply deserved. The best eulogy which vaccination can receive, is the countenance given to it by men of high and honorable principles, combined with a thorough acquaintance with the subject. From this combination it is, that we view *Dr. Ash's* eulogium as so highly creditable to *Dr. Jenner's discovery.*

---

The Monday evening Meetings of the *Medical Society of London* have commenced for the season, at the Society's house in Bolt-court.

---

The *Blenheim-street Medical Society* will resume its sessions on Saturday evening, November the 7th, at half past seven o'clock, at the Theatre of Anatomy in Blenheim-street.

---

The Meetings of the *Linnæan Society* will commence on the first Tuesday in November, in Gerard-street.

---

The *Westminster Medical Society*, held in Great Windmill-street every Saturday evening during the season, has recommenced its sittings.

---

In America, the oil of Turpentine has been applied in burns, especially in the unfortunate cases which occurred at the late conflagration of the Richmond Theatre. Most of the severe cases perished, and in those which recovered the stimulant applications proved extremely painful.

---

*Radical Cure of Hydrocele.*—A laboring man near Plymouth (New England) had long been afflicted with hydrocele of the tunica vaginalis testis, for which no remedy had been applied, and the tumor was of a large size. While he was standing near a vicious horse, the animal, being irritated, seized the patient by the scrotum with his teeth, in such a manner as to penetrate both the integuments and vaginal coat, by which the contents were immediately evacuated, and a radical cure was the happy result.—*New England Journal of Medicine and Surgery*, No. 2, April 1812.

---

Mr. Stevenson, author of the ingenious paper on Cataract, inserted in this and the preceding Journal, has recently had the honor of being appointed Occulist and Aurist to her Royal Highness Charlotte Princess of Wales.

---

We announce, with regret, the death of Dr. Edward Miller, editor of the New York Repository. He was affected, for some time, with a catarrh, which suddenly assumed the typhoid character, attended with delirium and extraordinary prostration of strength. After these appearances, he soon expired. In him the profession and the public have lost a valuable member; amiable and highly respectable in character, zealous in promoting professional improvement, ardent and strenuous in the cause of science.

*Statement*

*Deaths by Small-pox weekly, from the London bills.*

| | | | | |
|---|---|---|---|---|
| Sept. | 8, . . . . . | 27 | Sept. 29, . . . . | 34 |
| | 15, . . . . . | 24 | Oct. 6, . . . . . | 28 |
| | 22, . . . . . | 30 | | |

---

The Committee of Apothecaries, elected at a general meeting held by public advertisement on the 3d of July last, have prepared a Report relative to the present state of Pharmacy, and earnestly request the presence of every *Apothecary* who can make it convenient to attend at the Crown and Anchor, Strand, on Friday, November the 6th, at one o'clock, to take the same into consideration. For the very respectable names which compose this committee, see the advertisement on the cover of the Journal this month.

---

*Theatre of Anatomy, Bartlett's Court, Holborn.*—Mr. Armiger will commence his Lectures on Anatomy, Physiology, and Surgery, on the 2d of November.—Mr. A. proposes to give only one Course during the present season, and to arrange the Anatomical part of it into two series of demonstrations.

The first Series to comprehend the general Anatomy of the Human Body.

The second Series to show the relative Anatomy of the Seats of the most important Accidents, and of the Operations; thereby illustrating the application of anatomy to surgery, and the necessity of studying morbid structure.

The Physiological part of the Course will be limited to the Functions of the Human Body.

The Surgical part of the Course will comprehend general Observations on the Principles and Practice of the Art; and Demonstrations, 1st of the application of Means employed in Mechanical Surgery, and 2d of the Operations.

---

Mr. George Hodson, of Edinburgh, Ash-manufacturer, has obtained a patent for a Method of separating the Alkaline Salt from the Acid, as it exists in Kelp, Black Ashes, Soapers' Salts, Spent Leys, Soda, Natron, Rock Salt, Common Salt, Brine, Sea Water, Caput Mortuum of Aquafortis, Caput Mortuum of Oil of Vitriol, and Caput Mortuum of Salt used by Bleachers.

The following processes are stated by the patentee to be used for the purposes mentioned in the title of his patent:—A ton of kelp is ground to the size of a wallnut, and introduced into a vat or vats, with receivers under them, such as soap-boilers use. Water is then added, so as completely to cover it: after standing in that state twelve hours, the plug at the bottom is partially taken out, and the saline fluid drawn off in the usual manner soap-boilers follow. One hundred and sixty gallons of the first running are to be reserved, in order to mix with the remainder of the saline matter, to make the whole obtained of equal strength when it is introduced into the evaporating pan. The kelp in the vat should be covered with water, which should be kept running until the saline matter be wholly obtained. This saline matter is to be put into evaporating pans of a certain form, rather oval, each containing two hundred gallons more or less: five hundred weight of well-burnt lime, slaked and reduced to powder, and then immersed in water, in order that the stones and gravel may subside, must be added to the saline matter in proper proportions, to take in the whole of the saline matter, as it is introduced into the evaporating pans. Five hundred weight of charcoal, or cinders, or pit-coal, ground to dust, and afterwards put through a fine sieve, is also introduced into the evaporating pans, in proper proportions, along with the lime, and ten pounds of vegetable alkali, more or less, equally divided between the pans, which will accelerate the process. A strong fire is to be put under the pan, care being always taken that the fire is placed at a proper distance from the pan; and constant stirring must be employed until the lixivium begins to rise, which it will do in a similar manner to soap when boiling, and it must be treated by cooling as soap is, otherwise it will overflow the top of the pan. As the liquid evaporates, soft water must be added until the acid is dispersed, which will be effected in proportion to the quantity of saline matter in the pan with kelp of medium quality. The operation is finished in twelve or fourteen hours. Care must be taken not to fill the pan too full, in order to give the fluid liberty to rise. Care must also be taken frequently to stir the bottom of the pan with an iron stirrer, made in length in proportion to the depth of the pan. When the acid is found to be evaporated, which may be known by a simple chemical process upon a tea-cup full of the fluid in the pan, the fire must be partially drawn, and kept moderate, until the moisture is wholly evaporated. The fire must then be removed, and the substance in the pan stirred until it is cool, so as to prevent its adhering to the bottom of the pan. This substance is now in a state fit to be used by the soap-makers, in the same way they use other ashes, by bruising it small, and mixing it with quick lime, properly slaked. If a manufactory for preparing the ash for sale should be established, the substance must be put in a reverberating furnace, and such heat applied as to render it fluid; in which state it may be kept half an hour with a moderate fire. During the half hour when fluid, it is to be frequently stirred with an iron rake, made for the purpose. It may be stirred in the dry state a few times before it becomes fluid. It is drawn out of the furnace, in a fluid state, into an iron pot, placed under the door of the furnace. When cool, it comes out of the pot in a solid mass, similar in appearance to barilla.

The same mode of procedure must be followed with all the other substances in the patent. Those of them which contain earth in a state of combination, as the kelp does, must be bruised quite small, and dissolved in soft water; common salt must also be dissolved.

Another part of the process is to be conducted in a reverberating furnace; the kelp is to be bruised as the soap-makers now grind it for use, and five or six hundred weight of it is to be introduced into the furnace, along with two hundred weight of black peat moss, or earth, or moss made small, and moistened with water; fire is to be applied, applied, and with a proper iron rake it is to be stirred frequently, until the peat moss is consumed, which will be in six or eight hours; it is then to be drawn out, and is fit for use. All the other substances in the patent must be treated in the same manner.

The first process detailed by Mr. Hodson for separating alkalies from acids, in the salts in which they are united, would lead an inexperienced person to suppose that this was an operation of great facility; for he actually talks of its being effected before the application of the reverberating furnace; but those who try it, will not find it quite so easy as they might suppose. The addition of the lime, in the slaked state mentioned, in conjunction with charcoal, does not seem likely to be of any service in the process, the only effectual ingredient in which appears to be the vegetable alkali; and even that is directed to be used in a proportion much too small, to have any considerable effect on several of the salts mentioned.

The second process which is directed to succeed the first, is the most likely to be effectual, and appears to contain the only point of novelty in the specification; which is, the use of peat moss with the salts in the reverberating furnace. Some species of turf or peat (as that of Tingrith Moor) contain sulphate of iron, which, along with the carbonaceous matter of the peat, would convert some of the salts mentioned into sulphurets; which the continuation of the action of the reverberating furnace would ultimately reduce to their respective alkalies. In several situations also, the use of peat would be a very cheap method of supplying the carbonaceous matter wanted for the reduction of the salts; and therefore, in either point of view, its introduction appears the most valuable part of the patent: but, as the patentee has not secured the right of the use of it for all sulphates; since he has not given any directions for the previous reduction of any of the salts mentioned to the state of sulphates, one of the most valuable applications of the use of peat in the production of alkalies, appears to be left open to the free use of the public, which is the reduction by its means of sulphates in the manner stated; to which state all the salts mentioned, not of this description, might be brought by either the direct use of sulphuric acid, or by treating them in a proper manner with some other salt containing this acid in a weaker state of combination, than that which would be formed with it by the alkalies contained in the salts.

---

Mr. Andrew Patten, of Hulm, in the parish of Manchester, county of Lancaster, Iron-liquor Manufacturer; and Mr. Charles Hankinson, of Hale, in the parish of Bowden, in the county of Chester, Tanner; have obtained a Patent for a Discovery and Improvements in the Tanning of Leather by the use of Pyrolignous or Wood Acid.—Dated Jan. 1812.

Three methods are stated by the patentees for the application of pyrolignous acid to tanning. In the first method the hides and skins are to be first limed, haired, fleshed, and beamed, in the usual manner, to be well washed and cleared of the lime, and *masterings*, and then to be immersed in a pit of weak ooze or liquor made from oak bark, in which they must remain five or six weeks, and be handled well well till they begin to bloom; they are then to be taken out and immersed in a pit of pyrolignous or wood acid, having first purified it as shall be mentioned, for ten or fourteen days, according to the substance of the hides or skins, taking care to handle them well every day. If the hides or skins be light, the pyrolignous acid should be weakened, by putting to it an equal quantity of water or spent ooze; or three or four times as much water or spent ooze, if the hides or skins be very light: the strength of the liquor must be regulated by the lightness of the hides or skins, and they should be handled well every day till they be sufficiently raised; and then they should be immersed in a pit of clear water, or not, as convenient, and should remain in it one or two days, and be handled well every day. Then, in order to bring the hides or skins as near as possible to the color which is generally given to leather, they should be removed into pits of strong ooze, and be suffered to remain there for three or four weeks, or they may be put into two leys of bark, in the usual way of tanning, and remain in each three or four weeks, and then they may be taken up and dried for sale; but, if the hides be very heavy, it will be prudent to let them remain a little longer both in the pyrolignous acid and in the ooze.

In the second mode of applying the pyrolignous acid (after the hides or skins have been limed, haired, fleshed, beamed, and washed and cleaned from the lime and masterings, as before mentioned,) spent bark is to be taken, or fustick, or American bark, or shumac from which the strength has been drawn by the dyer, or any other mode of using it, or any other kind of spent wood, or ground wood, or saw-dust; and any of them is to be mixed with pyrolignous acid in a pit, in the same proportion in which oak bark is used to make a strong ooze. And into this the hides or skins are to be immersed, taking care to handle them well once each day; after lying in this pit one day, they are to be removed into another pit, in which any of the ingredients above mentioned is mixed with water, in the same proportion as with the pyrolignous acid; after lying in this pit one day, they are to be moved back again to the first pit, and this alternation of the pits is to be repeated every other day for six or eight weeks, according to the substance of the hides or skins, until they be sufficiently tanned, taking care to stir up the liquor in the pit well before the hides be put into it.

Light skins will be soon tanned in this manner, but they must be well attended to. Fifteen or twenty gallons of pyrolignous acid put into a pit of the spent ooze will renew it, and make it fit for receiving fresh hides.

In the third method, the pyrolignous acid is used alone, or only has the other articles added to it in the filtering or clearing, and very good leather may be thus made. Heavy hides not more than half tanned may be taken out of the bark ooze, and be immersed in the pyrolignous acid, taking care to handle them well, and they will be tanned through in two or three weeks, after which they may be taken out and put into a pit with a layer of bark, and be left there for two or three weeks until they be finished, and have a good color, when they are to be taken up and dried for sale: using the bark

 after after the pyroligneous acid, takes away the smell, and gives the leather a better color.

The method used by the patentees for purifying and clearing the pyrolignous acid from tar, and oily matter, is to heat a quantity of it in a pan, and immediately to throw over its surface a quantity of fine ground dry spent bark, taking care, as soon as the surface of the liquor is covered, to skim off all which lies on it: these operations are to be repeated, till about half a pound of the bark for every gallon of the liquor has been thrown in; and the skimming is to be continued till the liquor comes near boiling, and then the fire is to be drawn from under the pan, and the liquor emptied from it into a tub, or tubs, to cool and settle; after which it is fit for use.

To prevent any of the tanning principle from being lost, what is skimmed from the pan, and what has settled at the bottom of the tub, are to be mixed well with water sufficient to extract the remaining strength from them, and then are to be left to settle, when the clear liquor is fit for use. If the tanner prepares his own liquor, these skimmings and residua may be thrown into the leak pits, or spenders, which will make a stronger liquor for tanning than a pit of fresh ooze; and, for clearing the pyrolignous acid, spent fustick, spent shumac, spent American bark, ground wood, or saw-dust, if freed from color that might injure the leather; or ground pipe clay, or other clay or loamy earth, if it contain no metallic matter, may be used instead of the bark, for throwing over the surface of the liquor in the pan, to clear it as before mentioned; but the patentees prefer the bark: they have also used lime and whiting for the same purpose, and have found that with either the liquor retains a tanning principle, but has not the same effect as when cleared in the way before described.

As one of the methods proposed by the patentees for tanning, by which they assert very good leather may be made, is to use the pyrolignous acid by itself, their new process seems to bid defiance to all the former theories of tanning; in which a principle, contained in many substances, called tannin, was supposed to have an effect in hardening and making insoluble the gelatinous and other parts of skins, which converted them into leather. This should induce the patentees to lay before the public the strongest proofs, that leather prepared in their manner is as good as that tanned in the usual method; since otherwise much doubt will be entertained on this point, for the reason stated.

If it should be proved that pyrolignous acid really has the property of tanning leather by itself, it will be a most valuable discovery; and will add another to the many beneficial uses to which this acid is now applied, and give reason to suppose it may yet rival sulphuric acid in the extent of its application in the arts. It will also give us another cause to be thankful to the memory of the learned Glauber, who first recommended this acid to notice, and pointed out several of its valuable properties; some of which, not yet applied to profit, these discoveries may induce others to try on his recommendation.—*Repertory of Arts.*

