# Corrigendum

**Published:** 1812-12

**Authors:** 


### *CORRIGENDUM.*

In our last, p. 422, for *Rev. J. Nightingale and Rev. J. Andrewes, Curators*, read *W. C. Pettigrew and J. Andrewes, Esquires.*

